# An *In Vivo* Transfection Approach Elucidates a Role for *Aedes aegypti* Thioester-Containing Proteins in Flaviviral Infection

**DOI:** 10.1371/journal.pone.0022786

**Published:** 2011-07-27

**Authors:** Gong Cheng, Lei Liu, Penghua Wang, Yue Zhang, Yang O. Zhao, Tonya M. Colpitts, Fabiana Feitosa, John F. Anderson, Erol Fikrig

**Affiliations:** 1 Section of Infectious Diseases, Department of Internal Medicine, Yale University School of Medicine, New Haven, Connecticut, United States of America; 2 Department of Entomology, Connecticut Agricultural Experiment Station, New Haven, Connecticut, United States of America; 3 Howard Hughes Medical Institute, Chevy Chase, Maryland, United States of America; Institut Universitaire d'Hématologie, France

## Abstract

Mosquitoes transmit pathogens that cause infectious diseases of global importance. Techniques to easily introduce genes into mosquitoes, however, limit investigations of the interaction between microbes and their arthropod vectors. We now show that a cationic liposome significantly enhances delivery and expression of plasmid DNA in *Aedes aegypti* and *Anopheles gambiae* mosquitoes. We then introduced the genes for *Ae. aegypti* thioester-containing proteins (*Ae*TEPs), which are involved in the control of flaviviral infection, into mosquitoes using this technique. *In vivo* transfection of *Ae*TEP-1 into *Ae. aegypti* significantly reduced dengue virus infection, suggesting that the approach can further our understanding of pathogen-mosquito interactions.

## Introduction

Mosquitoes transmit pathogens of medical importance throughout the world. Approximately 700 million people develop mosquito-borne infectious diseases each year, and more than 2 million individuals die of these illnesses [1]. The ability of mosquitoes to transmit viruses and protozoa varies widely. Dengue virus (DENV) and Yellow Fever virus (YFV) are primarily transmitted by *Aedes aegypti* [2], [3]; West Nile virus (WNV) is carried by *Culex spp.* and *Aedes spp.* [4]–[6]; and the genus *Anopheles* is the dominant vector for *Plasmodium spp.* [7]. Approved human vaccines are not yet available for many of these infectious diseases [8]. An comprehensive understanding of the survival of microbes in mosquitoes may facilitate the development of new strategies to control transmission and reduce infection. The limitation of current approaches to introduce genes into mosquitoes, however, impairs efforts to investigate the interactions between pathogens and these important arthropod vectors.

Studies in mosquitoes currently rely on several techniques, including RNA interference (RNAi) [9] and germline transformation [10]. RNAi is the most popular approach, and has been broadly applied to functional studies in mosquitoes [11]. Another common strategy is to produce stable transgenic mosquitoes through germline transformation. There are, however, some drawbacks in these approaches. A serious concern about RNAi is that dsRNA/siRNA may promote off-target silencing, leading to ambiguous conclusions of gene function as it relates to microbial infection [12], [13]. Germline transformation is inefficient and time consuming, and the maintenance of transgenic strains is costly and laborious [14]. Development of a simple approach to efficiently express a gene in mosquitoes is, therefore, urgently needed and will accelerate the study of pathogen-mosquito interactions. We now report on the efficient introduction of plasmid DNA into mosquitoes and use this technique to elucidate the role of *Ae. aegypti* thioester-containing proteins (*Ae*TEP) in flaviviral infection.

## Results

**Figure 1:**
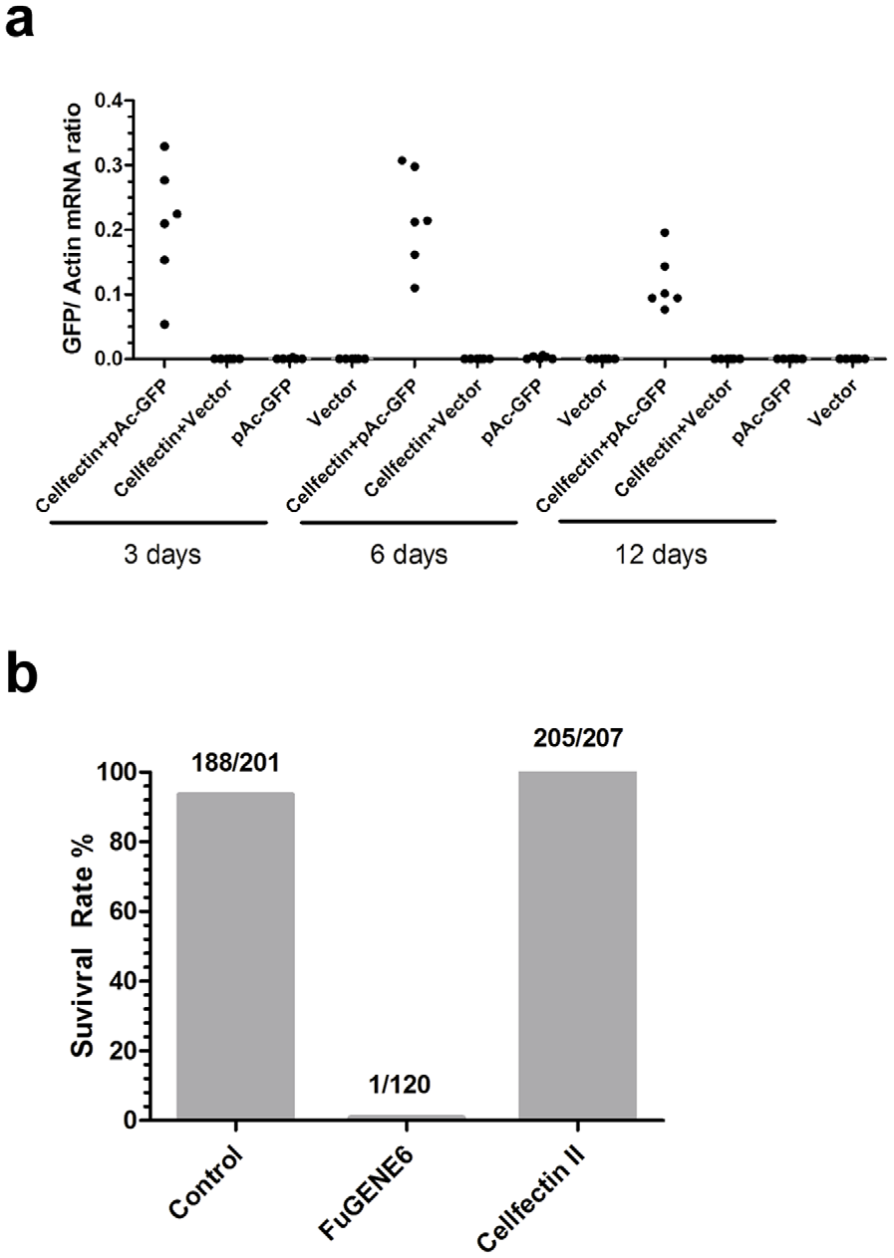
Cellfectin II® enhances *GFP* mRNA *in vivo* expression in *Ae. aegypti*. (**a**) Cellfectin II® significantly enhances *GFP* expression in *Ae. aegypti*. 200 ng pAc-GFP and the empty vector DNA were microinjected per mosquito. Total RNA was isolated from whole mosquitoes and the samples were decontaminated by Dnase I treatment. The *GFP* expression was determined by SYBR Green® QPCR, and normalized using *Ae. aegypti actin (AAEL011197)*. Each dot represents 1 mosquito. The experiment was repeated twice with similar results. (**b**) Cellfectin II® does not influence mosquito survival. Each liposome reagent mixed with vector DNA was microinjected into the mosquito (200 ng DNA each mosquito). The mosquito survival was recorded at 6 hours after materials inoculation. Control mosquitoes were treated with PBS. The number at the top of bar represents the number of live mosquitoes/total mosquitoes in a treatment.

An efficient approach to deliver genes into mosquitoes is a powerful tool for mosquito genetic studies. We used a GFP-expressing plasmid, pAc-GFP, which has a *Drosophila actin* promoter and *GFP* gene, to optimize the experimental condition. To transfect the *GFP* gene into mosquitoes, we directly microinjected pAc-GFP or empty vector DNA into the thorax of mosquitoes, as described in a previous study [15]. GFP expression, however, could not be readily detected either by RT-QPCR (Figure 1a) or by fluorescence microscopy (data not shown). This suggests that direct inoculation of DNA is inefficient and that protein expression is insufficient for functional studies.

### *In vivo* transfection of pAc-GFP plasmid with Cellfectin II®

A previous study showed that an exogenous gene was transfected into dissected *Ae. aegytpi* salivary glands by an *in vitro* liposome-based transfection [16]. We therefore reasoned that a cationic-liposome could also deliver plasmid DNA with high efficiency into live mosquitoes. Liposome-based reagents are widely used for DNA transfection into insect cell lines. Cellfectin II® [17] and FuGene 6® [18] are common transfection reagents for cellular studies. We therefore examined whether these reagents could deliver plasmid DNA into adult female mosquitoes with high efficiency.

**Figure 2:**
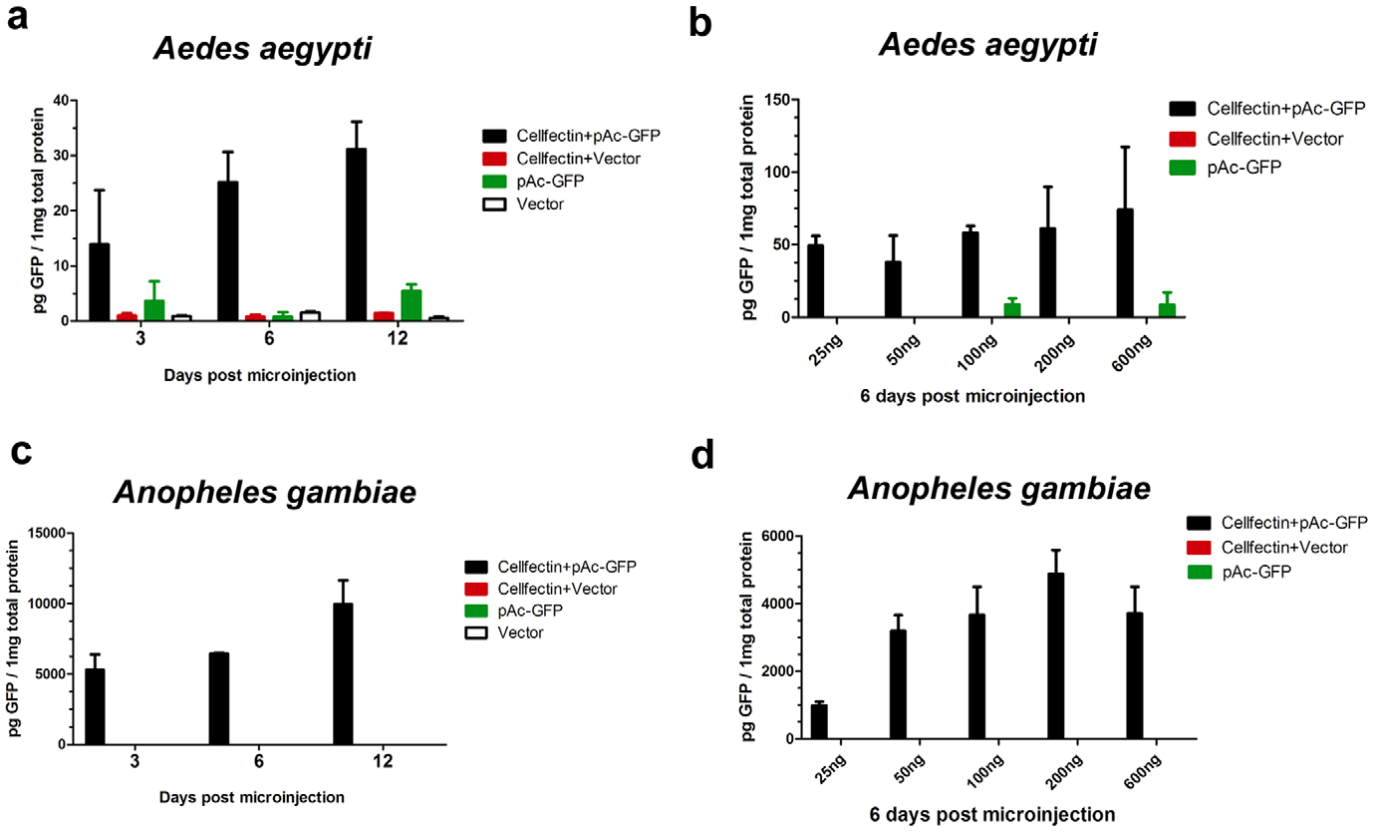
Cellfectin II® enhances GFP protein expression in *Ae. aegypti*. Cellfectin II® facilitates GFP expression in *Ae. aegypti* (**a**) and in *An. gambiae* (**c**). 200 ng plasmids were microinjected into mosquitoes. The GFP protein concentration in whole mosquito lyses was measured by ELISA. Data are expressed as the mean ± standard error from 3 independent experiments. Titration of the amount of plasmid DNA influences the GFP expression in *Ae. aegypti* (**b**) and in *An. gambiae* (**d**). 2-fold titrated plasmid DNA with Cellfectin II® was inoculated into mosquitoes. The treated mosquitoes were sacrificed at 6 days post microinjection and homogenated in tissue lysis buffer. The GFP protein level was determined by ELISA. Data are expressed as the mean ± standard error from 3 independent experiments.

Liposome is potentially harmful to mosquitoes. FuGene 6® was lethal to *Ae. aegypti*, as after thoracic inoculation, only 1 out of 120 mosquitoes survived. Cellfectin II®, however, was well tolerated by mosquitoes (Figure 1b), suggesting that it may be suitable for *in vivo* transfection studies. To evaluate the ability of Cellfectin II® to deliver plasmid DNA, we mixed Cellfectin II® with the pAc-GFP plasmid and microinjected the liposome-DNA mixture into *Ae. aegypti*. Empty DNA vector and pAc-GFP in medium served as controls. *GFP* mRNA was quantified by RT-QPCR at various time points. The empty vector and pAc-GFP alone groups expressed very little *GFP*; however, Cellfectin II® significantly enhanced *GFP* expression (Figure 1a). Consistent with the QPCR results, GFP protein was dramatically enhanced by Cellfectin II® post microinjection (Figure 2a). We then titrated the plasmid DNA via 2-fold dilutions, and microinjected this material with Cellfectin II® into mosquitoes. As seen in Figure 2b, a minimum of 25 ng pAc-GFP per mosquito produced substantial GFP expression in *Ae. aegypti*.

*Anopheles spp.* transmit *Plasmodium*, the etiologic agent of malaria [7], [19]. We therefore extended this approach to *An. gambiae*, and assessed whether this method was effective in another mosquito species that transmits important diseases. Cellfectin II® also significantly increased GFP expression in *An. gambiae* (Figure 2c). Moreover, the amount of GFP protein was 100–500 folds greater in *An. gambiae* than in *Ae. aegypti* (Figure 2c). In the titration analysis, GFP expression was dramatically decreased when the inoculated DNA was less than 50 ng per mosquito (Figure 2d). To determine the distribution of expression, GFP was assessed in mosquito tissues by microscopy. Fluorescence was observed in the *An. gambiae* salivary glands and midgut. Figure S1 shows fluorescence at days 3 and 6 post injection in the liposome-DNA treated groups, confirming that Cellfectin II® facilitates the *in vivo* expression of DNA in mosquitoes.

### *In vivo* overexpression of *Ae*TEP-1 influences flaviviral infection

The *Thioester-containing protein* (*TEP*) gene exists in numerous mosquito species, and forms a distinct clade of a multigene family. TEP includes several vertebrate complement domains, and is considered a complement-like protein in mosquitoes [20]. A *TEP* gene from *An. gambiae* mediates recognition and killing of *Plasmodium berghei* ookinetes [21]. In invertebrates, *TEPs* are a component of the innate immune system to pathogens. The role of *TEPs* in flavivirus infection is, however, unknown. We therefore determined the role of *Ae. aegypti TEP* (*AeTEP*) genes in DENV and WNV infection by RNA interference, and validated the phenotype by *in vivo* overexpression.

**Figure 3:**
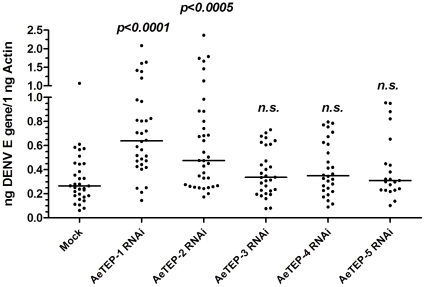
Silencing *AeTEPs* enhances DENV infection. *AeTEPs* were silenced by dsRNA treatment. The mock group was treated with the same amount of *GFP* dsRNA. The DENV burden was examined at 6 days post-infection. 10 MID_50_ DENV was used to challenge mosquitoes. The viral load was determined by Taqman® QPCR, and normalized by *Ae. aegypti actin*. Each dot represents 1 mosquito. The Mann-Whitney test was used for statistic analysis. The horizontal lines depict the medians of result. The result shown was combined with 3 independent experiments.

We identified 6 *TEP* homologues from the *Ae. aegypti* genome and designated them as *AeTEP-1* to *AeTEP-6*. *Ae*TEPs share 21–39% amino acid identity with *Anopheles* TEP-1. Of these 6 genes, 5 *AeTEP*s were expressed in the adult female *Ae. aegypti* (Table S1). *AeTEP*s genes were silenced in *Ae. aegypti* by gene-specific dsRNA. Compared to the mock group, expression of all the *AeTEP*s was significantly reduced from days 3 through 9 (Figure S2a, S2b, S2c, S2d, S2e). WNV and DENV were therefore inoculated into mosquitoes on day 3 following dsRNA-treatment, and the viral load on day 6 post-infection was quantified by RT-QPCR. The viral loads in *AeTEP-1* and *AeTEP-2* silenced mosquitoes were enhanced by 2–3 fold when compared with those of mock-treated mosquitoes (Figure 3 and Figure S3), suggesting that *Ae*TEPs may play an important role in resistance to flaviviral infection of *Ae. aegypti*.

**Figure 4:**
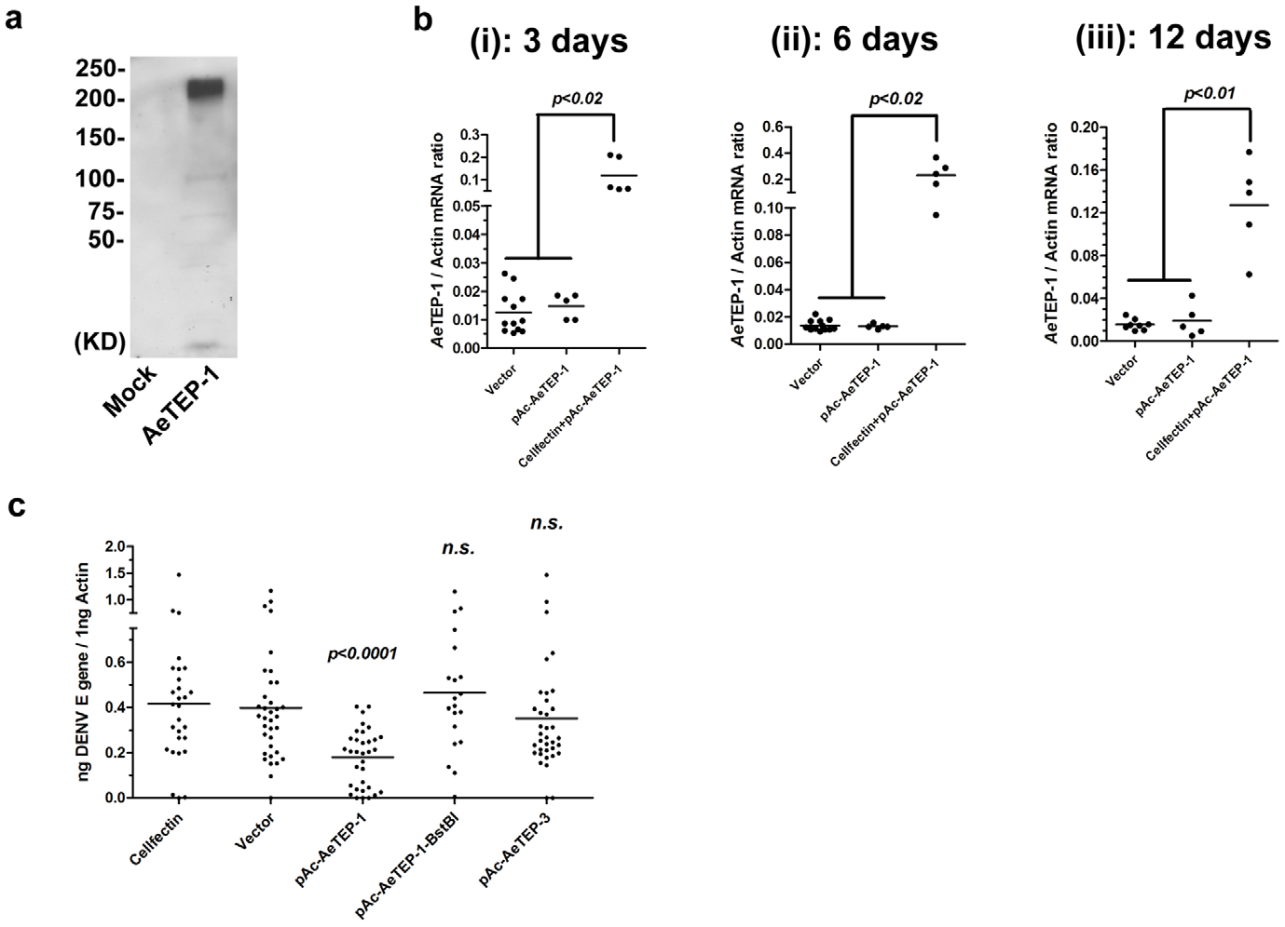
*In vivo* overexpression of *AeTEP-1* impairs DENV infection. (**a**) *Ae*TEP-1 expression in S2 cells. pAc-*Ae*TEP-1 expressed a ∼200 KD recombinant protein in *drosophila* S2 cells. *Ae*TEP-1 was detected with a V5-HRP mAb. The mock control was the empty vector transfected S2 cells. (**b**) Cellfectin II® facilitates *AeTEP-1* expression in *Ae. aegypti* at 3 days (i), 6 days (ii) and 12 days (iii) post microinjection. Controls are the mosquitoes inoculated with empty pAc vector or only pAc-*Ae*TEP-1 plasmid DNA in medium. The mRNA level of *AeTEP-1* was determined by SYBR Green® QPCR, and normalized using *Ae. aegypti actin*. The experiment was repeated 3 times with similar results. (**c**) Overexpression of *Ae*TEP-1 significantly decreased DENV burden. The viral load was determined by Taqman® QPCR, and normalized by *Ae. aegypti actin*. The result shown was pooled from 3 independent experiments. (**b and c**) Each dot represents 1 mosquito. The Mann-Whitney test was used for statistical analysis.

To further confirm the function of *AeTEP*s in flavivirus infection, we selected *AeTEP-1* and *AeTEP-3* for *in vivo* overexpression investigation. Meanwhile, a truncated *AeTEP-1* (*AeTEP-1-BstBI*) was generated as a control for functional study. These genes were cloned into pAc5.1/V5-His A vector and the recombinant plasmids were named as pAc-*Ae*TEP-1, pAc-*Ae*TEP-1-BstBI and pAc-*Ae*TEP-3 respectively. Then, these genes were expressed in *Drosophila* S2 cells. Immunoblot confirmed the expression of a ∼200 kD recombinant protein encoded by the *AeTEP-1* (Figure 4a) and ∼100 kD proteins by *AeTEP-3* and truncated *AeTEP-1-BstBI* genes (Figure S4a). Subsequently, these recombinant plasmids were microinjected into mosquitoes with Cellfectin II® to assess the efficiency of *in vivo* over-expression. The same amount of empty pAc vector or pAc-*Ae*TEP plasmids alone served as controls. The mosquitoes were collected at various time points after microinjection to isolate the total RNA for the detection of *AeTEPs*. *AeTEP-1* (Figure 4b) and *AeTEP-3* expression (Figure S4b) were significantly higher in the Cellfectin II® transfected groups than in controls, at all time points. Then, the *AeTEPs* transfection mosquitoes were used to elucidate the role in flaviviral infection. The Cellfectin II® alone and empty vector inoculated mosquitoes served as negative controls. Compared to the controls, the DENV burden was significantly decreased in the *Ae*TEP-1 expressing group (*p<0.0001*), but not in the *Ae*TEP-3 or *Ae*TEP-1-BstBI expressing groups (Figure 4c), suggesting *AeTEP-1* has a specific role against DENV infection. However, the overexpression of *Ae*TEP-1 did not influence WNV infection of mosquitoes (Figure S4c), implying that the *Ae*TEP-1 function is ambiguous in WNV infection. In conclusion, our results demonstrate the usefulness of this *in vivo* transfection strategy for functional study of mosquito genes.

## Discussion

Mosquitoes transmit numerous infectious diseases. Development of techniques to rapidly manipulate mosquitoes will facilitate our understanding of the pathogenesis of vector-borne diseases. Genetic studies on mosquitoes are generally conducted in cell-culture. These studies provide important information; however, understanding the pathogenesis of vector-borne microbes requires *in vivo* studies. Here, we used a transfection reagent to introduce plasmid DNA into mosquitoes through thoracic microinjection. This procedure enabled us to successfully express an important gene *in vivo*. Our results demonstrated that the method worked in both *An. gambiae* and *Ae. aegypti*, albeit better in *An. gambiae*. However, Cellfectin II® did not efficiently transfect plasmid DNA into *Ixodes scapularis* (data not shown), a tick that transmits diverse pathogens including the agent of Lyme disease. These data indicated that different arthropod species has varying sensitivity to this liposome-based transfection.

In this study, we assessed the antiviral function of the family of *Ae. aegypti* complement-like factors using RNAi and *in vivo* transfection approaches. The complement system combats viral infection in mammals [22]. Complement components are associated with viral surface proteins during early infection and are activated to promote the destruction of viral particles and initiate innate immune responses [23], [24]. In flaviviral infection, complement components can activate B cells, assist in B cell maturation, and enhance viral neutralization in an antibody-mediated manner [25]. Recent studies have shown that a complement-like factor, *Thioester-containing protein* (*TEP*) genes, exists in *An. gambiae* to mediate parasite killing [21], [26]. To determine the role of *TEP* genes in flavivirus infection, we identified five expressed *TEP* homologues in female *Ae.aegypti*. Silencing certain *AeTEP* by RNAi led to a significant enhancement of WNV and DENV burdens. To further confirm the role of *AeTEPs* in flaviviral infection, we chose *AeTEP-1* and *AeTEP-3*, and also generated an *AeTEP-1* truncation, for *in vivo* over-expression. Compared to liposome alone and vector controls, *Ae*TEP-1 expression dramatically decreased the DENV infection, however, the same phenomenon was not observed in *AeTEP-3* and truncated *AeTEP-1*-transfected mosquitoes. Together with the gene silencing results, we concluded *AeTEP-1* plays a role in resistance to DENV infection of mosquitoes. *Ae. aegypti* is a primary mosquito species for DENV infection and transmission in nature. Similar to mammals, mosquitoes also evolve specific defense mechanism, including complement-like factors, to efficiently control DENV infection. *AeTEP-1* silencing also showed a strong resistance in WNV infection. However, the *in vivo* transfection of *AeTEP-1* did not elucidate a similar phenomenon, suggesting *Ae*TEPs-based resistance system is important, but not essential in WNV infection.

In summary, we have developed an efficient method to express an exogenous gene in mosquitoes *in vivo*. The technique was validated by assessing the importance of an *Ae. aegypti TEP* gene in flaviviral infection. This method is potentially useful in mosquito-pathogen interaction studies in many ways: validation of RNAi silencing results, *in vivo* biochemical studies and mosquito genetic investigations. This approach should facilitate an understanding of the interaction between mosquitoes and the infectious agents that they transmit.

## Materials and Methods

### Mosquitoes, cells and viruses

*Ae. aegypti* and *An. gambiae* mosquitoes were maintained in a sugar solution at 27°C and 80% humidity according to standard rearing procedures [27], [28]. The *Aedes albopictus* C6/36 cell line was grown at 30°C in minimal essential medium (MEM) for WNV and DENV production. The *Drosophila melanogaster* S2 cell line was cultured in Schneider's Medium. All mediums were supplemented with 10% heat-inactivated fetal bovine serum, 1% L-glutamine, 100 IU of penicillin and streptomycin per ml. WNV strain 2741, which is identical at the protein level to the NY99 strain [29], and DENV-2 (DENV New Guinea C strain) were used in this study. The viruses for *in vivo* experiments were titrated in mosquitoes through thoracic microinjection. The procedure was described in a previous study [11].

### DNA construction

GFP gene was amplified from pCAG-GFP plasmid and subcloned into pAc5.1/V5-His A vector (Invitrogen, Cat. No# V411020). The recombinant plasmid was designated as pAc-GFP in this study. The cloning primers for pAc-GFP were: Forward, GCTACAGAATTCATGGTGAGCAAGGGCGAGGAG; Reverse, GCTACACTCGAGCTTGTACAGCTCGTCCATGC. *Ae. aegypti TEP-1* and *TEP-3*, including a signal peptide gene, was produced from a cDNA library and cloned into pAc5.1/V5-His A. The recombinant plasmid was named as pAc-*Ae*TEP-1 and pAc-*Ae*TEP-3 respectively. The cloning primers for pAc-*Ae*TEP-1 were: Forward, TCTGAGTTTAAACATGACCCGTCGGTGGCATCC; Reverse, TCTGACCATGGCTAATCGTTCCAAAAGATCC. The cloning primers for pAc-*Ae*TEP-3 were: Forward, GTACTCGGTACCATGTCCTACTTTGTCACAAAC; Reverse, GTACTCCTCGAGACAGCTCATCGATCTGCAATC. A truncated *Ae*TEP-1 in pAc vector, pAc-*Ae*TEP-1-BstBI, was constructed by BstBI restriction enzyme treatment. The BstBI digestion in pAc-*Ae*TEP-1 depletes the 2640 bp-5382 bp region of *AeTEP-1*, leading to an in-frame truncation.

### Cellfectin II® *in vivo* transfection

Cellfectin II® (Invitrogen, Cat No# 10362-100) was mixed with S2 Schneider's medium (serum/antibiotics-free) as 1∶1 ratio (vol/vol). The mixture was kept at room temperature for 10 min. 2 vols of the diluted plasmid DNA were then combined with diluted Cellfectin II® and gently mixed. The transfection mixture was further incubated for 30 min at room temperature prior to thoracic microinjection.

### Microinjection

dsRNA synthesis was performed as described previously [30]. The primers are shown in the Table S1. Plasmid DNA was isolated by an EndoFree Plasmid Maxi Kit (Qiagen, Cat. No# 12362). For thoracic microinjection, 1 week old adult female mosquitoes were kept on ice for 15 min, and then transferred to a cold tray to receive a systemic injection of dsRNA or plasmid DNA into the hemocoele. In the gene silencing studies, 2 µg of dsRNA/300 nl in PBS was microinjected into the thorax of each mosquito. Following a 3-day recovery period, the mosquitoes were microinjected with WNV or DENV 10 M.I.D_50_/300 nl (50% Mosquito Infective Dose) [11] for functional studies. In the *in vivo* transfection, 200 ng plasmid DNA/300 nl mixture was inoculated into each mosquito. The mosquitoes were further challenged by 10 M.I.D_50_ of DENV and WNV at day 6 following the inoculation of the transfection mixture.

### Quantitative PCR

The specific RNAs of the *WNV-E* gene, *DENV-E* gene and *AeTEPs* gene were quantified by RT-QPCR. The primers and probes for *WNV-E* and *DENV-E* gene were described previously [11]. The primers for *AeTEPs* are shown in Table S1. The amount of viruses were normalized using *Ae. aegypti actin* (*AAEL011197*).

### ELISA

Three mosquitoes were pooled together and homogenated in 300 µl T-PER tissue protein extraction buffer (Thermo Scientific, Cat. No# 78510) with a Pestle Grinder System (FisherSci, Cat. No# 03-392-106). The samples were centrifuged for 10 min at 4°C and the supernatant were collected for ELISA assay. The experimental details are described in the product manual of GFP ELISA kit (Cell Biolabs, Cat. No# AKR-121).

### Isolation and imaging of mosquito tissues

Salivary glands and midgut were dissected as previously described [31]. Tissues were isolated, placed on sialylated slides (PGC Scientific, Gaithersburg, MD), washed in PBS, and fixed in 4% PFA at 37°C for 1 hr. Slides were imaged with a Single-Track mode of a Zeiss LSM 510 meta confocal microscope.

## Supporting Information

Figure S1 **GFP fluorescence in mosquito tissues.** The tissues were dissected at day 3 and day 6 post microinjection. The fluorescence was detected by confocal microscopy. (**a**) salivary glands; (**b**) midgut. Images were examined using a Zeiss LSM 510 meta confocal 10×objective lens.
(TIF)

Figure S2 ***AeTEPs*** **RNAi efficiency.** The mock group was treated with the same amount of *GFP* dsRNA. *AeTEPs-dsRNA* or *GFP-dsRNA* treated mosquitoes were sacrificed to isolate total RNA at 3 days and 9 days post-inoculation. mRNA of *AeTEPs* was determined by SYBR Green® QPCR, and normalized using *Ae. aegypti actin*. (**a**) *AeTEP-1*; (**b**) *AeTEP-2*; (**c**) *AeTEP-3*; (**d**) *AeTEP-4*; (**e**) *AeTEP-5*. Each dot represents 1 mosquito. The Mann-Whitney test was used for statistical analysis.
(TIF)

Figure S3 **Silencing** ***Ae*** **TEPs enhances WNV infection.** *AeTEP* genes were respectively knocked down by dsRNA treatment. The mock group was treated with the same amount of *GFP* dsRNA. At 3 days post-silencing, 10 MID_50_ WNV was microinjected into mosquitoes. The WNV burden was examined at 6 days post-infection. The viral load was determined by Taqman® QPCR, and normalized by *Ae. aegypti actin*. Each dot represents 1 mosquito. The Mann-Whitney test was used for statistical analysis. The horizontal line depicts the medians of the result. The result shown is the combination of 3 independent experiments.
(TIF)

Figure S4 ***Ae*** **TEP-3 and** ***Ae*** **TEP-1-BstBI expression and the role of** ***AeTEPs*** **in WNV infection.** (**a**) *Ae*TEP-3 and *Ae*TEP-1-BstB1 expression in S2 cells. *Ae*TEPs were detected with a V5-HRP mAb. The mock control was the vector transfected S2 cells. (**b**) Cellfectin II® facilitates *AeTEP-3* expression in *Ae. aegypti* at 6 days (i) and 12 days (ii) post microinjection. Controls are the mosquitoes inoculated with empty pAc vector or only pAc-*Ae*TEP-3 plasmid DNA in medium. mRNA of *AeTEP-3* was determined by SYBR Green® QPCR, and normalized using *Ae. aegypti actin*. The experiment was repeated twice with similar results. (**c**) The *Ae*TEPs *in vivo* expression in WNV infection. The viral load was determined by Taqman® QPCR, and normalized by *Ae. aegypti actin*. Each dot represents 1 mosquito. Statistical analysis used the Mann-Whitney test.
(TIF)

Table S1 **AeTEP family in** ***Ae. aegypti*** **.**
(DOC)
