# Supplementary figures and images for: An In Vivo Transfection Approach Elucidates a Role for Aedes aegypti Thioester-Containing Proteins in Flaviviral Infection
Source: PLoS One. 2011 Jul 27;6(7):e22786. doi: 10.1371/journal.pone.0022786 (PMC3144946; doi:10.1371/journal.pone.0022786)

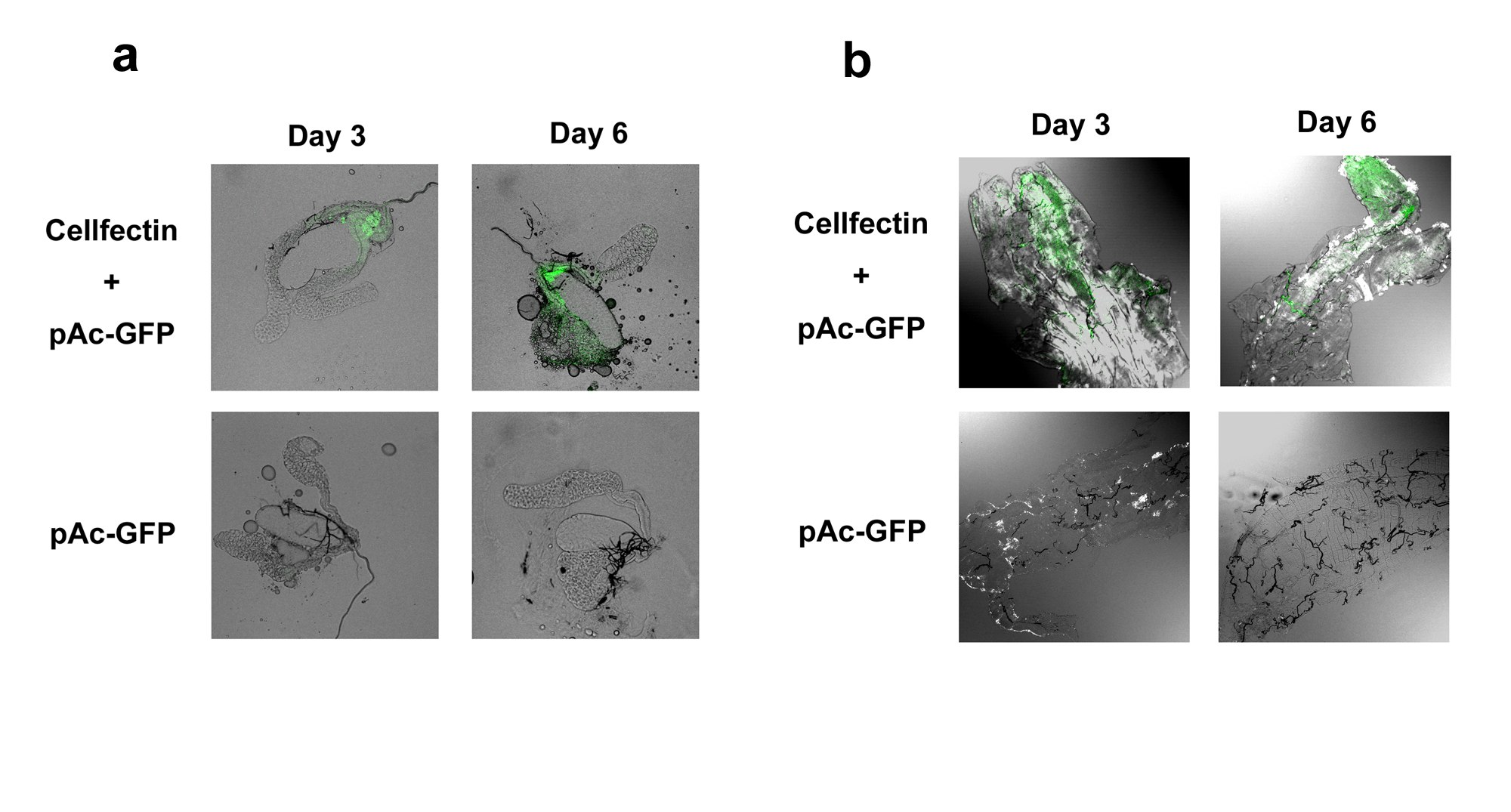

Supplement: Figure S1 — GFP fluorescence in mosquito tissues. The tissues were dissected at day 3 and day 6 post microinjection. The fluorescence was detected by confocal microscopy. (a) salivary glands; (b) midgut. Images were examined using a Zeiss LSM 510 meta confocal 10×objective lens. (TIF) [file pone.0022786.s001.tif]

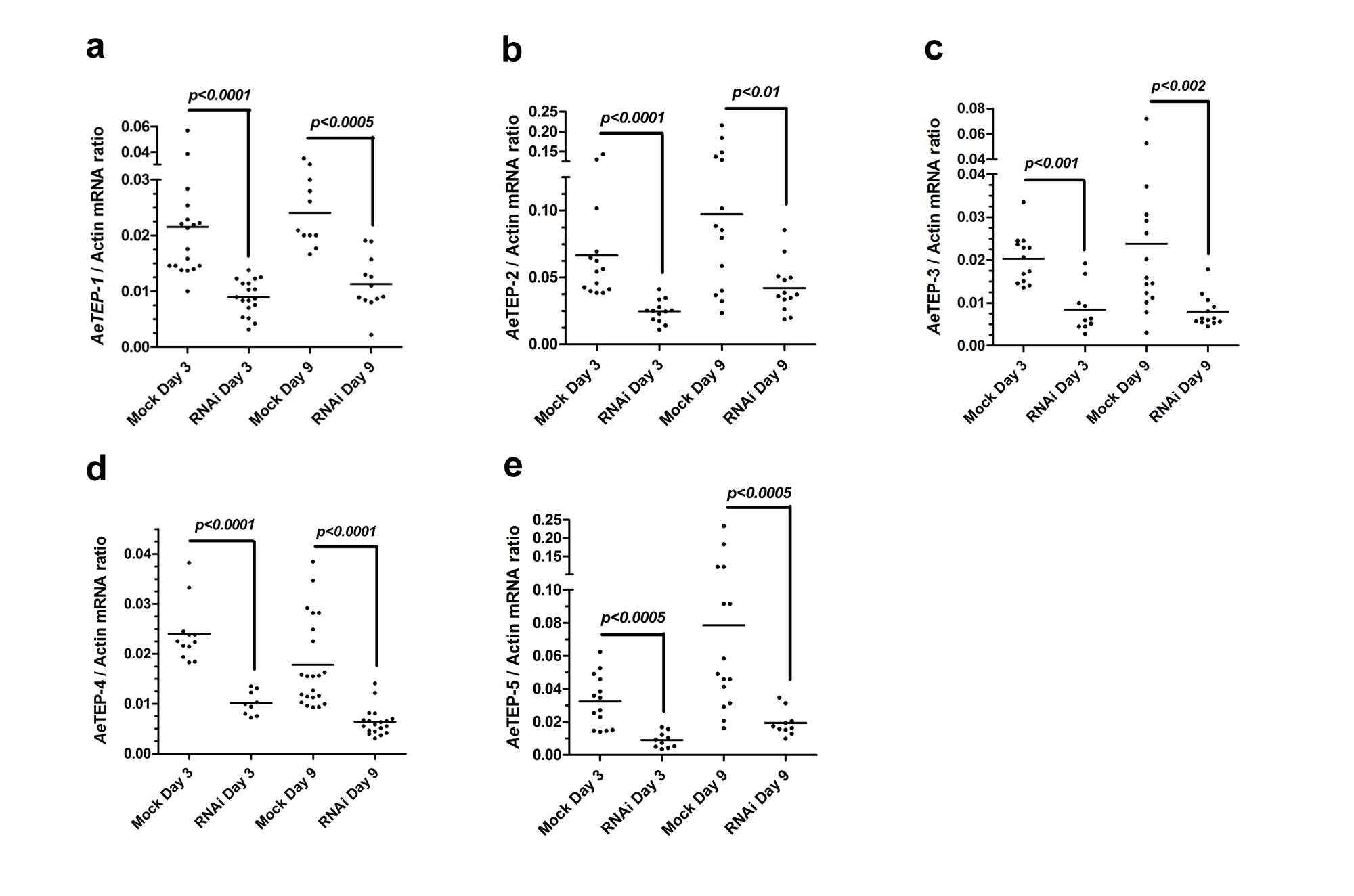

Supplement: Figure S2 — AeTEPs RNAi efficiency. The mock group was treated with the same amount of GFP dsRNA. AeTEPs-dsRNA or GFP-dsRNA treated mosquitoes were sacrificed to isolate total RNA at 3 days and 9 days post-inoculation. mRNA of AeTEPs was determined by SYBR Green® QPCR, and normalized using Ae. aegypti actin. (a) AeTEP-1; (b) AeTEP-2; (c) AeTEP-3; (d) AeTEP-4; (e) AeTEP-5. Each dot represents 1 mosquito. The Mann-Whitney test was used for statistical analysis. (TIF) [file pone.0022786.s002.tif]

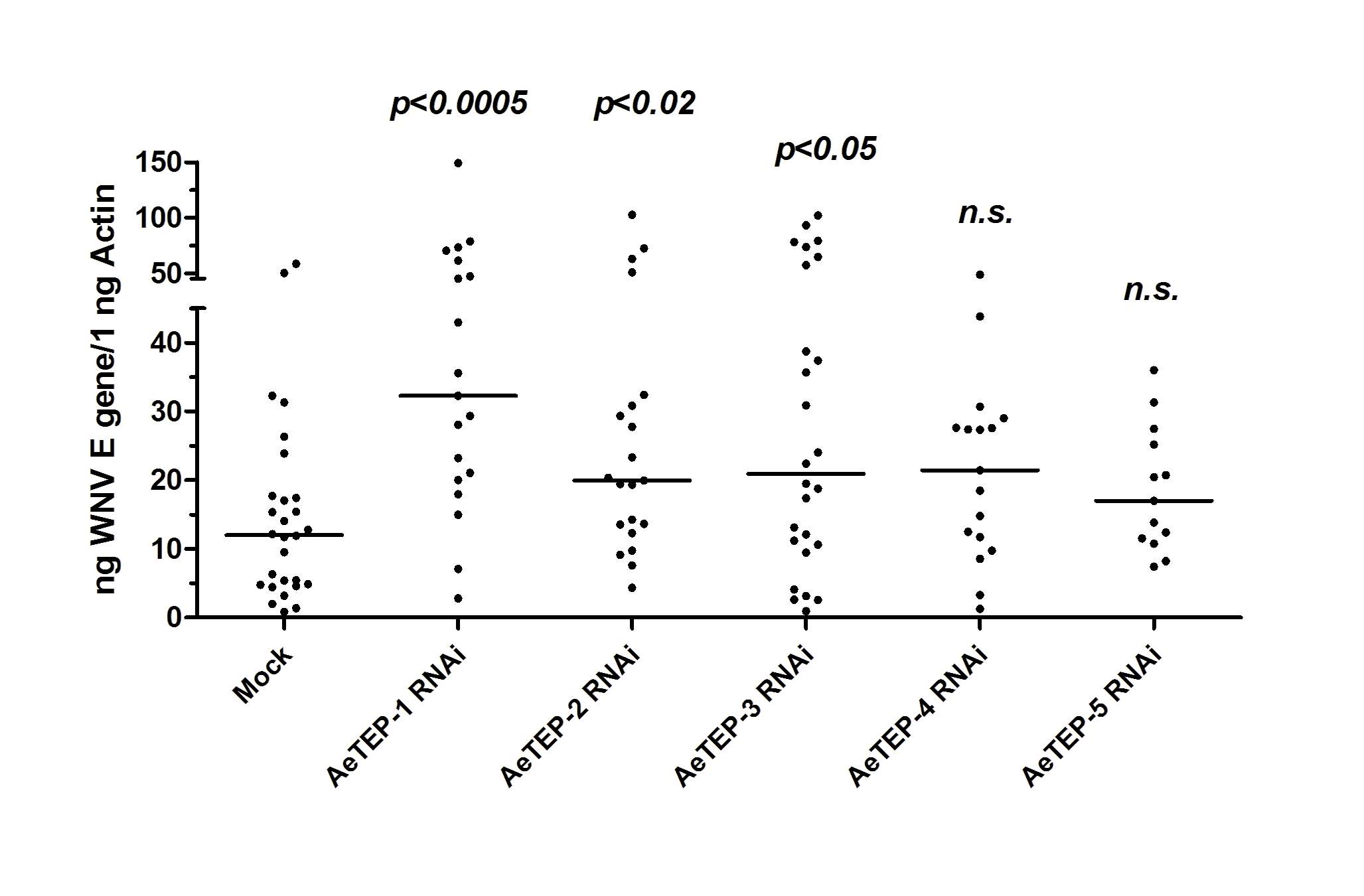

Supplement: Figure S3 — Silencing Ae TEPs enhances WNV infection. AeTEP genes were respectively knocked down by dsRNA treatment. The mock group was treated with the same amount of GFP dsRNA. At 3 days post-silencing, 10 MID50 WNV was microinjected into mosquitoes. The WNV burden was examined at 6 days post-infection. The viral load was determined by Taqman® QPCR, and normalized by Ae. aegypti actin. Each dot represents 1 mosquito. The Mann-Whitney test was used for statistical analysis. The horizontal line depicts the medians of the result. The result shown is the combination of 3 independent experiments. (TIF) [file pone.0022786.s003.tif]

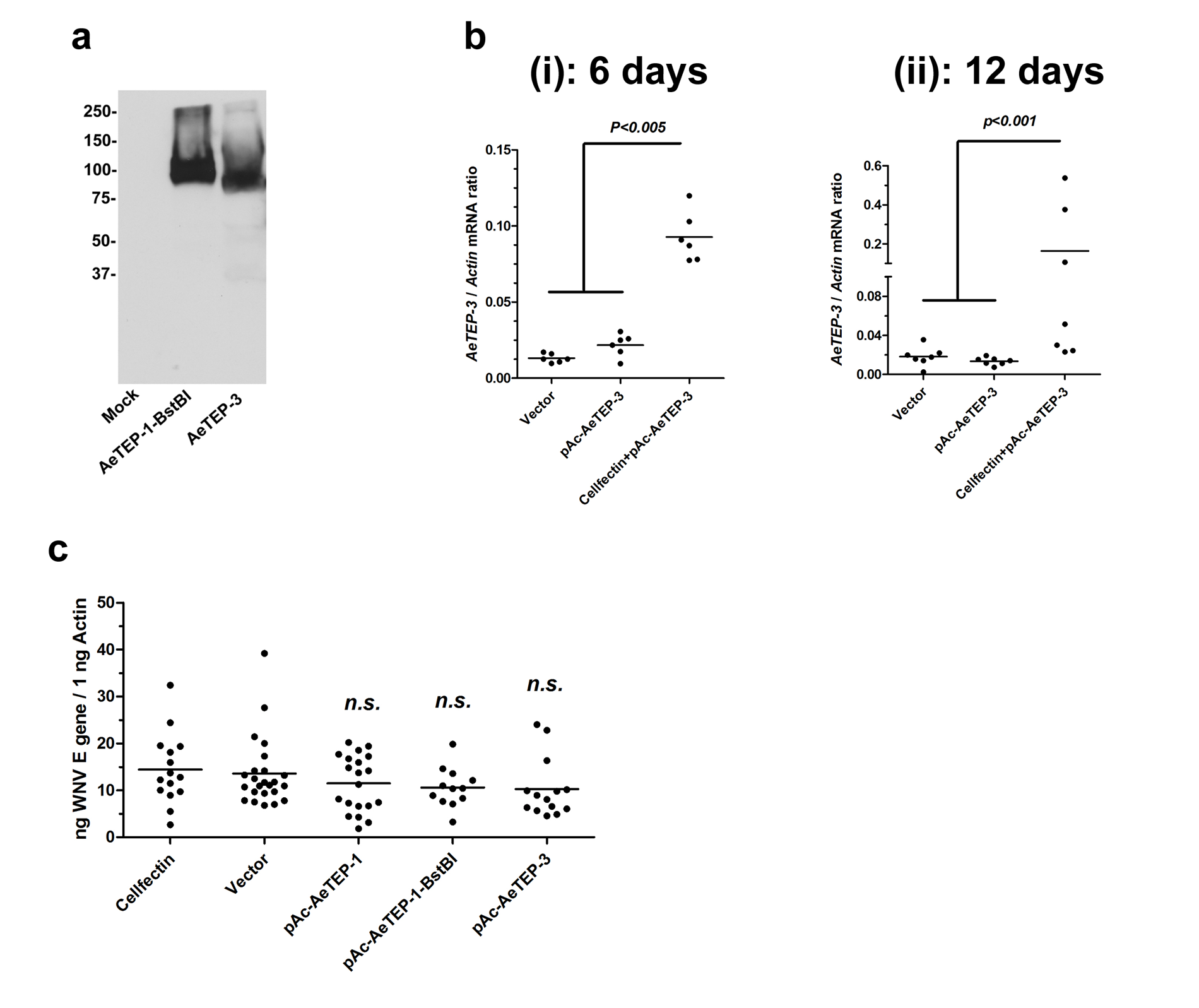

Supplement: Figure S4 — Ae TEP-3 and Ae TEP-1-BstBI expression and the role of AeTEPs in WNV infection. (a) AeTEP-3 and AeTEP-1-BstB1 expression in S2 cells. AeTEPs were detected with a V5-HRP mAb. The mock control was the vector transfected S2 cells. (b) Cellfectin II® facilitates AeTEP-3 expression in Ae. aegypti at 6 days (i) and 12 days (ii) post microinjection. Controls are the mosquitoes inoculated with empty pAc vector or only pAc-AeTEP-3 plasmid DNA in medium. mRNA of AeTEP-3 was determined by SYBR Green® QPCR, and normalized using Ae. aegypti actin. The experiment was repeated twice with similar results. (c) The AeTEPs in vivo expression in WNV infection. The viral load was determined by Taqman® QPCR, and normalized by Ae. aegypti actin. Each dot represents 1 mosquito. Statistical analysis used the Mann-Whitney test. (TIF) [file pone.0022786.s004.tif]
