# Supplementary material for: An In Vivo Transfection Approach Elucidates a Role for Aedes aegypti Thioester-Containing Proteins in Flaviviral Infection
Source: PLoS One. 2011 Jul 27;6(7):e22786. doi: 10.1371/journal.pone.0022786 (PMC3144946; doi:10.1371/journal.pone.0022786)
Supplement: Table S1 — AeTEP family in Ae. aegypti . (DOC) [file pone.0022786.s005.doc]

| Gene Symbol | GenBank No. | Identity to *An*TEP-1 **^b^** | Expression **^c^** | dsRNA Primers | QPCR Primers |
| --- | --- | --- | --- | --- | --- |
| *Ae*TEP-1 | AAEL012267 | 21.0% | Yes | TAATACGACTCACTATAGGGGGAGGCCGCAACACATACGAC | TCCGTCCCGGTCAGATTTACAA |
|  |  |  |  | TAATACGACTCACTATAGGGCGATTCCCGACAAACCCACATA | TCTACGCCGTCACGGGAGATAC |
| *Ae*TEP-2 | AAEL014755/ AAEL008067**^a^** | 33.2% | Yes | TAATACGACTCACTATAGGGCTGGCCGGAGATGGTGTTGAG | AAACGGGGAGAGGTGGTGTCG |
|  |  |  |  | TAATACGACTCACTATAGGGCCGCCTTGCATATCCGTGTG | CGTCGGCAAACTCAAACTCCTG |
| *Ae*TEP-3 | AAEL000087 | 35.8% | Yes | TAATACGACTCACTATAGGGTCGCACGAAGGCCATCATAGC | ACGGGGCGTTTGGTCAATG |
|  |  |  |  | TAATACGACTCACTATAGGGCGCCCCGTTCGAGTGTTTGTA | GGATGCCGTGGCAAGAGCA |
| *Ae*TEP-4 | AAEL001163 | 28.7% | Yes | TAATACGACTCACTATAGGGCTGACGGTCGCGGAAGTGTATT | CCTGGCTTTGGACGGAAATGA |
|  |  |  |  | TAATACGACTCACTATAGGGACGCGAGTATTTTCCCAGTGCT | TCCCAGCCCATGATTCGTGTT |
| *Ae*TEP-5 | AAEL001794 | 39.1% | Yes | TAATACGACTCACTATAGGGAACCAACACAGCGGCAGAAGAT | AATGCGGGTGGAAGTCGTAGG |
|  |  |  |  | TAATACGACTCACTATAGGGGAAAGCCTCCGAGACCATAACG | TTCCGTCGTGGTGTTGAGTTGA |
| *Ae*TEP-6 | AAEL001802 | 38.6% | No |  |  |
|  |  |  |  |  |  |

**Supplementary Table 1. AeTEP family in *Ae. aegypti*.**

^a^ AAEL008607 is 609 amino acids (aa) shorter than AAEL014755 in the N-terminal. The sequences from 610 aa to the end share 100% identity between AAEL008607 and AAEL014755.

^b^ *An*TEP-1, *Anopheles gambiae* Thioester-containing protein-1 (AGAP010815).

^c^ *Ae*TEP expression in the whole body of *Ae. aegypti*.
